# Genotoxicity Studies in Groundwater, Surface Waters, and Contaminated Soil

**DOI:** 10.1100/tsw.2002.338

**Published:** 2002-05-08

**Authors:** Luc Verschaeve

**Affiliations:** Vito, Environmental Toxicology, Boeretang 200, B-2400 Mol, Belgium

**Keywords:** Ames test, VITOTOX test, umu-C test, comet assay, comparative investigations, groundwater, surface water, soil

## Abstract

It is at present considered important to include biological tests in measuring programmes of environmental samples to supplement the chemical and physical parameters that are currently used. A battery of tests is therefore necessary, also within a given “endpoint” (e.g., genotoxicity), because one single test will not give all the answers to our questions. As it is not possible to include all available tests in routine screening programmes, a selection of tests should be made. According to comparative investigations, the bacterial Ames test remains very important. When no preconcentration step is involved, other bacterial tests (e.g., the umu-C and VITOTOX tests) may be recommended. The comet assay may be used in Daphnia or human white blood cells. Further validations, comparisons, mechanistic investigations, etc. remain necessary as differences are often found between the tests that are not solely explained by differences in genetic endpoint and that therefore should further be investigated and understood.

## INTRODUCTION

Time and budgetary limitations exclude the use of a large battery of genotoxicity tests for routine screening of environmental samples. Therefore, a limited number of tests that are technically simple, standardised, inexpensive, fast, ecologically representative, and reproducible are necessary. Bacterial tests are certainly among the recommended tests because they meet most of above-mentioned requirements. Among them the "classical" Ames test may be envisaged, but other tests can be applied as well. Such tests are, for example, the umu-C test, SOS-chromotest, VITOTOX® test, and others. Although DNA is universal and results obtained in bacteria may be more or less predictive for genotoxicity in higher organisms, including man, it may be important to perform also one or a few tests on eukaryotic organisms. One of the few tests that may be envisaged for routine screening is the alkaline comet assay. Contrary to cytogenetic methods (investigations of chromosome aberrations, sister chromatid exchanges, micronuclei, unscheduled DNA synthesis, etc.), this test can be performed on virtually any cell type, does not require proliferating cells, large cell populations, or labelling techniques, and allows a cell-by-cell investigation and hence the detection of intercellular differences[1]. Comparative investigations were performed in the past using these and other tests for analysis of pure chemical compounds as well as different environmental samples including surface and groundwaters, effluents, and others[2,3,4,5,6,7,8,9,10]. The literature data show that different tests may have different outcomes in different investigations, with different preferences and recommendations at certain times.

We further investigated a number of candidate tests for routine screening of environmental samples, not to identify the "best" test, but in order to determine which tests could be envisaged as part of a limited time and cost sparing battery of tests.

## EXPERIMENTAL METHODS/PROCEDURES

In the present paper, attention will be focused on the bacterial Ames test, umu-C test, and VITOTOX® test, as well as on the comet assay on earthworm coelomocytes, human white blood cells, and cells from *Daphnia magna*. However, where possible, comparisons with other tests are briefly made. The interested reader can find more details in the cited references. The Ames test detects reverse mutations in the histidine operon and was performed according to well-known standard methods using *Salmonella typhimurium* strain TA98[11]. The umu-C test was performed according to ISO/FDIS 13829 international standard guidelines[12] and the VITOTOX® test according to Verschaeve et al.[13] Both the umu-C and VITOTOX® test are based on error-prone DNA repair (SOS induction). Fusion of a reporter gene to a gene that is induced by the SOS-machinery following DNA damage is the key element in these tests. In the umu-C test, a fusion of a LacZ gene to SfiA results in synthesis of β-galactosidase when SfiA is transcribed following DNA damage. This can be visualised by colorimetric or photometric means. In the VITOTOX® test, a LuxCDABE gene from *Vibrio fisheri* is fused with a mutated recN gene resulting in rapid light emission following DNA damage. It can be recorded online using a luminometer. The comet assay was performed as described by Singh et al.[14], with small modifications according to the cell type and organisms used. Further details on the tests can also be found in Corbisier et al.[15].

## RESULTS AND DISCUSSION

During a recent BIOSET workshop[15], 11 genotoxicity tests were performed on pure chemicals and environmental samples. Among them, five bacterial tests were conducted (SOS chromotest, umu-C test, combined SOS and Lac Fluorotest, fiber optic RecA-lux test, and VITOTOX® test). All tests gave similar results and no false positives were identified. However, the SOS chromotest did not designate 2-aminoanthracene (2-AA) as genotoxic at a concentration of 4 μg/ml that was detectable in the other tests. Also, effluent waters from a textile industry appeared nongenotoxic in the SOS chromotest, while it was genotoxic in the other tests. Interestingly, the Laarbeek surface water in Zwijndrecht (Belgium), which is considered a "polluted river," was found to be nongenotoxic in most bacterial tests but genotoxic in higher target organisms (yeast, fish embryos, and human white blood cells). Overall, in this comparative investigation where crude samples were analysed without preconcentration or extraction, the VITOTOX® test and umu-C test were considered the most adequate bacterial tests. The Ames test reacted as expected, but is more time consuming. Maybe a modified Ames test, e.g., the Ames microsuspension version[16], can be advisable.

Water samples were also investigated in a research project from RIWA (an association of waterworks in the Netherlands)[17] to set up a test battery that is as suitable as possible. Here, surface water from the Meuse and Rhine Rivers were investigated following an XAD-25000 times preconcentration step at pH = 2 and pH = 7. In their research programme, different bioassays were used to evaluate and measure the presence of toxic compounds in rivers. They include a battery of toxicity and genotoxicity tests, as well as so-called effect-specific tests, as for example the acetylcholine esterase test to demonstrate the presence of organophosphates and carbamate pesticides, or the CALUX test to detect specific substances that may disrupt the hormone system in animals and humans. Among the genotoxicity tests the Ames test, umu-C test, VITOTOX® test, SOS-chromotest, MUTATOX test, and others were used. The comet assay was also conducted in human white blood cells and in *Daphnia*. All samples were positive in the Ames test, followed by the VITOTOX® test and umu-C tests. The comet assays clearly scored less well and the *Daphnia* comet assay was the least performing of all in terms of dose-effect relationship (dilutions), etc. Significant differences related to the rivers, acidity, and use of a metabolising S9 fraction were also observed. In the Ames test and the comet assay with leukocytes, a difference between the Meuse and the Rhine was found. The Ames test gave a lower response for the Rhine than for the Meuse, while the comet (leukocyte) test gave the opposite result. With regard to the use of the S9 liver extract, the Ames test turned out to yield a higher response when the S9 mix was added to the extract. The VITOTOX® test, on the other hand, gave more positive results in the extract without S9. The comet test with leukocytes responded mainly to hydrophilic compounds (pH 2), while the Ames test detected more mutagenicity on hydrophobic compounds (pH = 7). In terms of mutagenicity in the course of time, only the Ames test gave enough positive results to gain a picture of the evolution of mutagenicity over time.

Many differences were found indicating that it is necessary to better understand and analyse the mechanisms involved and the real significance of the results. For example, the comet assay in *Daphnia* is apparently less performing than the comet assay in human white blood cells. In the *Daphnia* test, a pool of cells is studied, some of which may have a higher or lower sensitivity than others. In addition, the cell population largely consists of a quickly proliferating population and hence cells are found in different stages of the cell cycle; this may influence the results[18]. In human white blood cells, a synchronized nonproliferating cell population is investigated. The advantage of the use of two comet tests is that the white blood cells have a clear link to humans, while the *Daphnia* test is more directly related to animals living in the water. The latter therefore gives more information about the ecological effects. That the Ames test scored better than the umu-C and VITOTOX® tests may eventually indicate that there were fewer compounds in the extracts that evoked an SOS response in the bacteria than those that only generated frame shift mutations (TA98), but at this point we just don't know. Also, *Salmonella* and *E. coli* are very efficient nitro-reducers. Thus nitro-containing chemicals may be efficiently converted to electrophilic species by bacteria and not by mammalian cells for which reductive metabolism is usually inhibited by oxygen. Many nitroso-containing compounds are therefore positive in bacteria but negative in mammalian cells.

Thus, the overall picture obtained is rather confusing. For the time being, and before alternative tests are further evaluated and validated, one may consider that the Ames test is still the most reliable. It demonstrates mutagenicity most frequently and also yields better doseresponse curves and the clearest results. When samples were subjected to a preconcentration or extraction procedure, high toxicity often prevented the correct evaluation of the genotoxic potential of the sample in the VITOTOX® test. It is our experience that the same holds true for the umu-C test. Other extraction or concentration procedures can be used to circumvent this problem. Alternatively, one could get rid of the solvents, e.g., by evaporation and resuspension of the extract in DMSO, but volatile genotoxins may then be eliminated. For effluents or samples that do not require a preconcentration or extraction procedure, the umu-C test or VITOTOX® test may be recommended as shown earlier.

We also performed the comet assay in earthworms for investigations of soil pollution. We believe that this organism is particularly well-suited for that purpose as cells (coelomocytes) can be obtained easily using an extrusion protocol[19] and the earthworms can be directly placed into the test sample.

Our earthworms are kept in our own breeding facility in a "standard" black earth. This is mixed with the soil to be tested. We do that because a test sample can be too toxic for the earthworms or have a very different physico-chemical composition that may influence the results. We therefore usually perform two "dilutions," one with a 70% test sample and one with a 25% test sample. A pilot investigation was conducted on soil samples collected in the vicinity of a coke oven area in Slovakia[20]. Two kinds of "control" samples were included: an "external control" and an "internal" control (standard black earth). DNA damage was mostly induced in earthworms brought in soil taken at 400 m from the coke oven but in the direction of prevailing winds. There was also a "dose-dependent" effect, and internal and external controls gave very similar results. This example just shows that the earthworm comet test is apparently able to detect the presence of genotoxins in soils and to discriminate between more or less contaminated sites.

Another example is given by an investigation of PAH-contaminated soil samples (unpublished data). Earthworms in PAH-contaminated soil samples had higher DNA damage than in control samples; however, in this case there was no dose-effect relationship, at least not when benzo(a)pyrene was taken as the most representative pollutant. This is something that is often found. The comet assay in earthworms, but also in other organisms and man, is able to show the presence of and exposure to genotoxins, but is not always able to demonstrate a dose-effect relationship. However, this is easily explained by the presence of other compounds (pollutants) in the test samples that were not measured. In our example, benzo(a)pyrene was certainly not the only contaminant in the soil.

The above-mentioned comparative investigations do demonstrate that the different tests that are based on different molecular induction mechanisms may logically give different results (see Fig. 1 for an example). It is therefore not possible to identify the best test to evaluate genotoxicity in a number of environmental compartments such as surface waters, soils, and groundwater. A *best test* simply does not exist. Comparisons of different tests are therefore only indicative of which tests could reasonably be used in rapid screening programmes with a maximal sensitivity and specificity. Overall, the Ames test remains a very important tool, but tests like the umu-C and VITOTOX®tests may certainly be considered, especially when no pre-concentration step is required. The comet assay on eukaryotic cell systems may be important as well, but it should be realized that *DNA comets* may result from different biological interaction mechanisms and conditions (including, for example, cell cycle stages and apoptosis) and is subject to phenomena that should be well-understood and controlled.

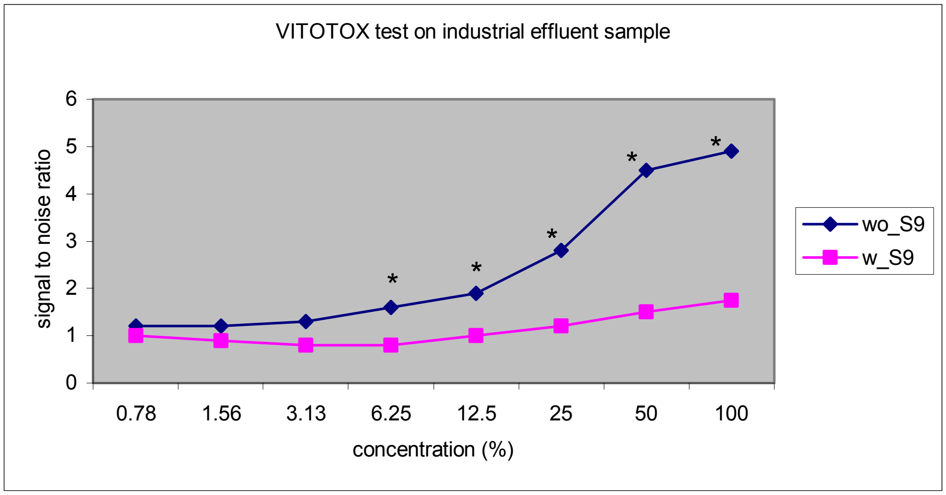

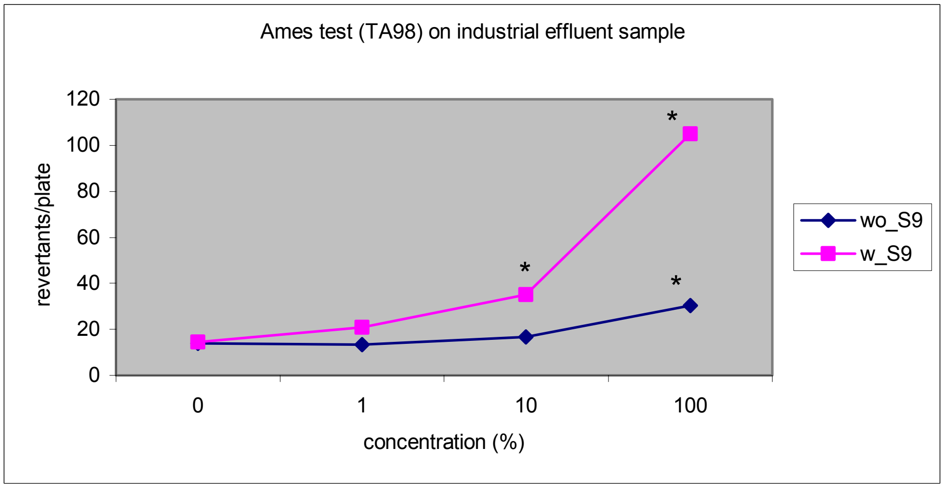

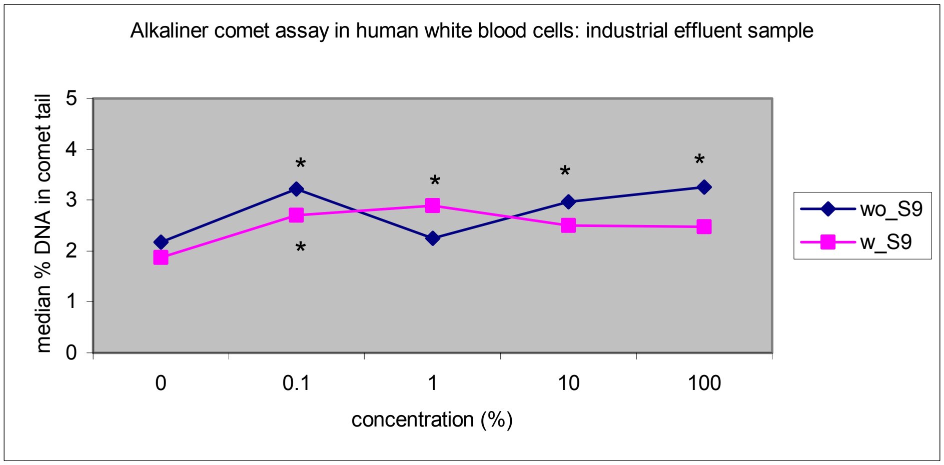

**FIGURE 1.** Genotoxicity testing of the same sample from an industrial effluent (textile industry) in three short-term tests. All three tests were able to recognize the sample as genotoxic; however, unlike in the Ames test and the comet assay, the VITOTOX® test only showed genotoxicity in the sample without addition of a metabolising S9 fraction. In the Ames test, the sample with addition of S9 was more effective in inducing mutations, whereas this was the opposite in the VITOTOX® test. The comet assay did not show a clear dose-response relationship. wo_S9 and w_S9 = resp. without and with addition of a metabolising rat liver S9mix; * = significant genotoxic effect; Concentration (%) = undiluted sample (100%) and dilutions up to 0.1% were added to the test organisms or cells.

## BIOSKETCH

**Luc Verschaeve** is Project Leader and Project Coordinator for Environmental Toxicology. He holds a Ph.D. in genetic toxicology. His research interests lie in the field of environmental toxicology, especially genotoxicology, with regard to water, air, and soil pollution. This also includes biomonitoring of humans/animals living in polluted regions. Another research activity involves the genetic and epigenetic effects of non-ionising radiation, e.g., mobile phone frequencies and extreme low-frequency fields (high- tension power lines). Genetic toxicology tests are also used for screening of chemicals, detection of irradiated food, and determination of mutagenic and anti-mutagenic properties of plant extracts used in traditional medicine.
